# Editorial: New trends in typical and atypical language acquisition

**DOI:** 10.3389/fpsyg.2025.1573869

**Published:** 2025-02-26

**Authors:** Eliseo Diez-Itza, Victoria Marrero-Aguiar, Alejandra Auza, Eva Aguilar-Mediavilla

**Affiliations:** ^1^LOGIN Research Group, Department of Psychology, University of Oviedo, Oviedo, Spain; ^2^Actualing, Clinical Applications, Technological and Related Uses of Linguistics, Department of Spanish Language and General Linguistics, National University of Distance Education (UNED), Madrid, Spain; ^3^Language and Cognition Laboratory, Hospital General Dr. Manuel Gea Gonzalez, Mexico City, Mexico; ^4^Investigation in Development, Education and Language (I+DEL), Institute for Research and Innovation in Education (IRIE), University of the Balearic Islands, Palma, Spain

**Keywords:** child language, language acquisition, phonetics and phonology, morphosyntax, lexicon and semantics, pragmatics and discourse

## 1 Introduction

This Research Topic presents a selection of contributions linked to the IX AEAL *International Congress on Language Acquisition*. The *Association for the Study of Language Acquisition* (AEAL; https://aeal.eu) promotes research on language acquisition and development in both, monolingual and multilingual contexts, with a particular focus on Spanish, Basque, Catalan and Galician, as well as the relationships between language and psychological, social, educational and biological processes. The triennial AEAL Congress has reached its 10th edition since 1995, and it has become one of the most relevant international scientific events in the field of language acquisition, bringing together experts in diverse areas, including grammar, lexicon, discourse analysis, pragmatics, psycholinguistics, neurolinguistics, sociolinguistics, and language teaching and education. A substantial body of research on language acquisition from these broad and interdisciplinary perspectives has been published in previous AEAL conference volumes (Pérez-Pereira, 1996; Mayor et al., 2005; Diez-Itza, 2008; Aguilar-Mediavilla et al., 2019).

The 18 articles included in this Research Topic provide an updated contribution to this research area, fostering and giving continuity to the dissemination and open discussion of new trends in the study of typical and atypical acquisition, as promoted by AEAL. They address the key topics covered in the AEAL Congress on Language Acquisition, including studies on phonological, morphosyntactic, and lexical-semantic levels, the development of discourse and pragmatics, literacy acquisition and development, language acquisition in bilingual and multilingual contexts, assessment and intervention in developmental language disorders, language learning and teaching, and new methodological approaches.

Accordingly, this Research Topic offers an integrated view of theoretical, methodological and applied issues from multilingual and multidisciplinary perspectives. It includes review articles, brief research reports and original research articles employing experimental, cross-sectional and longitudinal designs that cover a wide range of crucial issues in the field. The studies examine language acquisition among native speakers of various languages and dialects (i.e., Chilean, Colombian, Mexican and Peninsular Spanish; Basque; Catalan; Italian; English); in both monolingual and bilingual participants, across different age groups (infants, children, adolescents, and adults), and in both typical and atypical development (e.g., Developmental Language Disorder, Autism Spectrum Disorder; Williams and Down Syndromes, Hearing Impairment). A variety of research methods and assessment tools for spontaneous and elicited oral and written language are employed, including those from the CHILDES Project (MacWhinney, 2000), PROESC (Cuetos-Vega et al., 2002), PREP-CORP (Diez-Itza et al., 2022), PDP-PI (Junquera and Zubiauz, this volume), MacArthur Bates CDI (Fenson et al., 2007), SSRT Repetition Task (Bravo et al., this volume), The Pragmatics profile (Dewart and Summers, 1995), and MUAQ (Aparici et al., this volume). Additionally, instruments such as eye-tracking, the preferential-looking paradigm, multimodal input in foreign language teaching or technology-assisted intervention in neurodevelopmental disorders can also be found.

## 2 Typical language acquisition

The significance of preverbal abilities during the first year of life as a foundation for language emergence has been increasingly recognized over recent decades through diverse experimental paradigms. In this context, the study by Marcet et al., employing an eye-tracking preferential-looking paradigm, explore how monolingual and bilingual infants form face-language associations during their first year of life.

The development and use of diverse assessment tools and instruments in the early stages of language acquisition are essential to detect language difficulties and factors influencing language acquisition, a focus of several studies in the present volume. Bravo et al. examine the potential of a Spanish Sentence Repetition Task for detecting language disorders in young children, comparing it with measures of spontaneous language (Mean Length of Utterance, lexical diversity, and structure of the Noun Phrase). Ezeizabarrena et al. provide a brief review of the adaptation of the short MacArthur-Bates Communicative Development Inventories (CDIs) for Basque, designed to measure young children's vocabulary size. They also present data from a large sample assessed through the Basque CDI on the effects of age, sex, and language input in early language developmen. Junquera and Zubiauz introduce a new protocol for assessing the development of pragmatic competence in early childhood (PDP-PI), based on taxonomies of communicative functions (Interactional, Referential, Subjective and Figurative), along with preliminary data from its application in 3–5-year-old children. Botana and Peralbo investigate the influence of parental beliefs about child development and other socio-familial variables on the pragmatic assessment of infants and young children using The Pragmatic Profile, underscoring the need for contextualized evaluation.

Research on grammar comprehension has traditionally received less attention due to its methodological complexity. The work of Torrens investigates preschool children's comprehension of object and subject relative clauses using a cloze test, comparing cases with identical or different morphosyntactic features to evaluate sentence processing predictions based on the Relativized Minimality hypothesis. Another topic involving language comprehension, the effects of input, is addressed by Alvarado et al. who examine how preschoolers' oral narrative retelling is influenced by the source text, specifically on the production of conceptual subordination, as evidence of an interpretive process beyond simple reproduction of input.

Bilingual acquisition and its relatioships with literacy development are also central issues at AEAL conferences. Aparici et al. describe a newly developed Multilingual Use Assessment Questionnaire (MUAQ), administered to bilingual Catalan-Spanish children and young adults to capture the heterogeneous nature of multilingual profiles based on self-assessment of language competence, language use in mental operations, and language use in different contexts. Leonetti Escandell examines by means of a cloze-test and a written narrative retelling task the reference production in Italian-Spanish bilingual children, investigating whether it is influenced by referential expression type or language dominance. Finally, Feijoo and Anglada analyze how multimodal input, particularly gesture, contributes to the development of morphological awareness in English-speaking adolescents learning Spanish as a second language after short-term training with different input modalities.

## 3 Atypical language acquisition

Language acquisition research increasingly focuses on differentiating developmental trajectories to improve early diagnosis of atypical development and design personalized interventions, a trend reflected in several studies in this volume. Auza-Benavides et al. use the MacArthur-Bates Communicative Development Inventory II to investigate the variability in early expressive vocabulary among typically developing children, late talkers, and children at risk for neurodevelopmental conditions like autism spectrum disorder (ASD), and developmental language disorder (DLD).

A frequent cause of atypical language acquisition is Developmental Language Disorder (DLD), a primary language disorder that persists into school age and beyond. This impairment is investigated in two experimental studies utilizing eye-tracking. Guerra et al. examine lexical-semantic processing in preschool children with DLD, focusing on their real-time comprehension of semantic relationships, to verify expected difficulties in lexical access and retrieval, as well as greater lexical competition among children with DLD. Lara-Díaz et al. investigate visual attention during phonological processing tasks, also testing language, vocabulary, and phonological awareness in Colombian children with DLD to assess its role in integrating visual perceptual information with diverse cognitive and linguistic processes. Children with DLD not only face challenges in oral language but also show significant writing difficulties. This aspect is explored by Balboa-Castells et al., who utilize a writing process evaluation battery (PROESC) to analyze how these children plan and code written expository texts, examining word frequency and sentence structure, grammatical complexity, lexical density, as well as omissions and errors.

Two contributions in this volume stem from the SYNDROLING Project, which aims to identify specific linguistic phenotypes in neurodevelopmental genetic syndromes, as postulated by neuroconstructivist models. These studies employ the methods of language corpus analysis provided by the CHILDES Project. Martínez et al. explore the profiles of late phonological development of children with Williams syndrome, focusing on the absolute frequency of phonological errors, in a longitudinal analysis that tracks an accelerated evolution from expansion to stabilization stages following non-linear trajectories. Viejo et al. explore the pragmatic profiles of adolescents with Down syndrome and Williams syndrome by comparing the microstructure and macrostructure of their narratives, highlighting atypical dissociations, using the Pragmatic Evaluation Protocol for Corpora (PREP-CORP) to assess productivity and complexity at both levels.

The relationships between language and cognition in children with hearing loss have been recurrently addressed in developmental research, as they show significant delays in understanding Theory of Mind. This has often been attributed to limited access to conversational interactions in their environment. In this vein, Serrat et al. assess the connection between language development and mind-reading abilities in hearing-impaired children, and specifically whether the successful completion of a second-order false-belief task requires the comprehension of complements or other language skills, such as expressive vocabulary, receptive and expressive syntax, recalling sentences, and a recursive sentential complement.

New trends in the research on atypical language acquisition increasingly focus on the development of innovative intervention methods that incorporate technological devices. Finally, Urrea et al. present a systematic review, preregistered in PROSPERO, evaluating the effectiveness of technology-assisted interventions— using tablets and computers— for vocabulary learning in children with autism spectrum disorder, emphasizing essential factors such as personalized assessments, recognition of prior experiences, and awareness of the context of usage.

## 4 Conclusion

In sum, the articles included in this Research Topic provide a rich tapestry of interdisciplinary perspectives in the study of language development, encompassing a wide array of topics. The studies presented illustrate the dynamic and evolving nature of research on typical and atypical language acquisition, incorporating a broad range of theoretical and applied perspectives. A salient feature of this volume is its methodological diversity, employing experimental, cross-sectional, and longitudinal designs to investigate language acquisition across various age groups and linguistic backgrounds. Furthermore, the focus on bilingual and multilingual contexts reflects the growing recognition of the need to understand language acquisition in increasingly diverse linguistic environments. This Research Topic contributes to a deeper understanding of the mechanisms underlying language production, comprehension and processing and demonstrates the field's commitment to advancing reliable assessment strategies. The contributions also emphasize the need of early detection and effective intervention in atypical language development, highlighting the results of interdisciplinary collaboration and innovative methodologies in addressing the complexities of language development. This Research Topic underscores the relevance of AEAL's mission in shaping future directions in language acquisition research.
